# Ultrasonographic assessment of skeletal muscles after experimentally induced neurogenic inflammation (facet injury) in rats

**DOI:** 10.1177/15353702221119802

**Published:** 2022-09-13

**Authors:** Bahareh Ahmadi, Sara Issa, Felipe CK Duarte, John Srbely, Pawel M Bartlewski

**Affiliations:** 1 Department of Biomedical Sciences, Ontario Veterinary College, Guelph, ON N1G 2W1, Canada; 2 Department of Research and Innovation, Canadian Memorial Chiropractic College, Toronto, ON M2H 3J1, Canada; 3 Department of Human Health and Nutritional Sciences, College of Biological Sciences, University of Guelph, Guelph, ON N1G 2W1, Canada

**Keywords:** Rat, neurogenic inflammation, skeletal muscles, ultrasound image analysis, first-order echotextural variables, inflammatory regulators

## Abstract

This study set out to examine ultrasonographic attributes of non-neurosegmentally (pectoral-forelimb) and neurosegmentally linked (hindlimb) myotomes in an experimental model that leads to neurogenic inflammation in segmentally linked myotomes, and to evaluate quantitative correlations among ultrasonographic attributes of the muscles, relative content of various inflammatory mediators, and nociceptive thresholds (hot and mechanical) in rats. Twelve male Wistar Kyoto rats were randomly divided into two equinumerous groups: surgery group, in which the left lumbar (L4–L6) facet joints were compressed for 3 min with modified Kelly forceps under general anesthesia, and sham-operated rats. All ultrasonograms were obtained with the Vevo 2100 Visual Sonic scanner connected to a 24-MHz transducer at four different time points: pre-surgery and 7, 14, and 21 days after surgical procedures. Digital ultrasonographic images of quadriceps femoris, hamstring, and pectoral-brachial muscle groups were analyzed using a polygonal meter region of interest placed on the largest cross-sectional area of the muscles displayed in Image ProPlus^®^ analytical software to compute numerical pixel values and pixel heterogeneity (standard deviation of mean pixel values). On day 21, pain behavior tests (hot plate and von Frey) were performed and then all animals were euthanized. Protein expression of inflammatory mediators in biceps brachii and rectus femoris muscles was measured by Western blot. The most prominent differences in muscle echotextural attributes between the two subsets of rats occurred 14 days post-surgery in pectoral-brachial and quadriceps femoris muscles. The expression of calcitonin-gene-related peptide was directly related to both echotextural variables only in biceps brachii (pixel intensity: *r* = 0.65, *P* = 0.02; and heterogeneity: *r* = 0.66, *P* = 0.02, respectively). Our findings have revealed the occurrence of echotextural changes in skeletal muscles of rats during myositis; however, the accumulation of inflammatory mediators and the outcomes of sensory tests did not relate to the changes in first-order echotextural characteristics of affected hindlimb muscles.

## Impact Statement

In this study, we performed an assessment of skeletal muscles and pain threshold during experimentally induced neurogenic inflammation in rats and examined echotextural attributes of segmentally linked and segmentally uncoupled muscles with biochemical (levels of inflammatory regulators) and nociceptive thresholds (thermal and mechanical). Computer-assisted analyses of digital ultrasonograms have revealed the occurrence of echotextural changes in various skeletal muscles, both in sham-operated and experimental animals. Different muscles within the same segments of myotomes exhibited dissimilar patterns of changes in pixel intensity and heterogeneity, which warrants further studies of histomorphological factors responsible for differences in skeletal muscle echotexture. Moreover, the levels of inflammatory mediators and the results of sensory testing did not relate to the changes in first-order echotextural attributes of affected hindlimb muscles. The present findings will contribute to advances in computerized ultrasonographic analysis for studying structural changes in skeletal muscles associated with an array of pathological conditions.

## Introduction

Neurogenic inflammation of the skeletal muscle is a pathophysiological process in which specific neurotransmitters are released directly from the terminals of sensory/nociceptive nerves to trigger an inflammatory reaction including erythema, swelling, temperature increase, tenderness, muscle weakness, and pain.^1,2^ It has been shown that lumbar facet joint osteoarthritis, the most common cause of facet joint pain,^3^ is associated with neurogenic inflammation developing within neurosegmentally linked skeletal muscles of rats (i.e. hindlimb)^4^. Previous studies also revealed that certain neurogenically mediated pro-inflammatory peptides such as substance P and protease-activated receptor 2 (PAR2) accumulated in the neurosegmentally linked myotome following facet injury.^5^ These regulators of inflammation initiate the cascade of processes including perivascular, epimysial, perimysial, and endomysial infiltration of immune cells with accompanying edema in the acute phase, and generation of residual inflammatory by-products or fibrosis in the subacute or chronic stage of inflammation.^4,6^ Accumulation of inflammatory mediators in skeletal muscles may lead to considerable changes in their microstructure and chemical composition.^7^

Clinical decision-making leading to appropriate treatment and management of inflammatory muscle disease depends on accurate diagnosis. The standard diagnostic method in inflammatory muscle disease is muscle biopsy to perform histological examination and determine the amount of neurogenic peptides.^8^ However, this method is invasive and permits the evaluation of only a small fraction of a muscle at a time. Magnetic resonance imaging (MRI) of skeletal muscles is a non-invasive approach and can encapsulate the entire muscle or even multiple muscles simultaneously, but it is expensive and usually less likely to be immediately available. Although serum creatine kinase is a helpful indicator in both diagnosis and management of muscle inflammation, it is not specific for myositis and its measurements may show substantial individual fluctuations or variability.^9,10^

Computer-assisted, grayscale ultrasound image analysis can aid in the diagnosis of myopathies by recording quantitative changes in first-order echotextural characteristics (pixel intensity and heterogeneity).^11^ There is a great deal of evidence to suggest that computerized analysis of ultrasonograms is a useful, non-invasive, and non-destructive tool to detect physicochemical changes in monitored organs and tissues (e.g. human dystrophic muscles,^12,13^ ram testes,^14^ and chicken pectoralis major muscle)^15^. Several studies have been performed to determine the efficacy of ultrasound imaging as a method of diagnosing muscle inflammation. In 1993, Reimers *et al.*^16^ compared the diagnostic value and sensitivity of ultrasound technique with that of serum creatine kinase activity and electromyograms for detecting acute and chronic idiopathic inflammatory myopathies in adults. They confirmed that morphological basis for alterations in muscle ultrasonograms (i.e. edema, fat accumulation or fibrosis detected with histopathological examinations) was reflected in muscle sonographic features. In 2009, Fujikake *et al.*^17^ investigated whether the infiltration of inflammatory cells due to injection of bupivacaine hydrochloride to the tibialis anterior muscle of rats would alter echointensity of B-mode ultrasonographic images, but their study failed to support the null hypothesis suggesting that other inflammatory responses, such as edema, are primarily responsible for changes in muscle echotexture. In another study, however, it has been shown that calprotectin (protein released by neutrophils in inflammation) is associated with the sum scores from a comprehensive ultrasound assessment in rheumatoid arthritis patients.^18^ Based on these collective observations, it is feasible that ultrasound technique combined with computer-assisted analysis of ultrasonograms could provide a useful technique for assessing changes in biochemical composition of neurogenically mediated inflammatory conditions of skeletal muscle.

Neurogenic inflammation is commonly associated with somatosensory changes that are detectable using quantitative sensory testing techniques assessing mechanical pain threshold and allodynia.^4,5^ The electronic von Frey test and heat stimuli^19^ are commonly used to evaluate pain response in both humans and animals. Punctate mechanical allodynia and hyperalgesia can be measured using a pinprick or monofilaments of varying forces applied to affected dermatomes (e.g. plantar aspect of the hindpaws). Heat hyperalgesia can be similarly tested using a hot plate to quantify heat pain threshold.^19^ Previous research has not investigated the correlation between ultrasonographic imaging, inflammatory profiles and/or pain sensitivity behavior in animals with muscles affected by neurogenic inflammation.

The main goal of this study was to document quantitative changes in mean pixel intensity and heterogeneity of skeletal muscles, both neurosegmentally linked and non-neurosegmentally linked, and to examine them for associations with inflammatory mediators and pain response results recorded after lumbar (L4–L6) facet injury. We hypothesized that (1) after experimentally induced spine osteoarthritis at the L4–L6 spinal level in rats, significant differences would exist between the mean pixel values and heterogeneity of neurosegmentally linked muscles (i.e. quadriceps and hamstring) and non-neurosegmentally linked pectoral-forelimb muscles; and (2) significant correlations would exist between echotextural variables, neurogenically mediated peptides (substance P, calcitonin-gene related peptide (CGRP) and pro-inflammatory markers (PAR2, extracellular signal-regulated protein kinase (ERK1/2), and Ca²^+^/calmodulin-dependent protein kinase II (CaMKII)), and behavioral pain threshold scores.

## Materials and methods

### Animals and surgical procedures

Twelve male Wistar Kyoto rats aged approximately 4 months were housed (2–3 per cage) in a room with a 12-h alternating light–dark cycle and a stable temperature (23.0 ± 1.0°C), and fed pellet diet *ad libitum*. All invasive procedures performed in this study were approved by the Animal Care Committee of the University of Guelph (Guelph, ON, Canada). Animals were randomly divided into two equinumerous groups: experimental surgery or facet injury (animals underwent facet compression at segments L4–L6 on the left side), and sham-operated (animals with left side facets L4–L6 exposed but without facet compression). The surgical intervention employed to induce the lumbar facet injury has previously been used to induce cartilage degeneration as evidenced by the presence of both tactile primary and secondary allodynia as well as spinal cord neuroinflammation. Although the extent of inflammation varies with the cause of the injury and the ability of the body to repair and overcome the tissue damage, our general objective was to induce acute/subacute inflammation lasting approximately 2–6 weeks.^20^ A non-steroidal anti-inflammatory drug Carprofen (Rimadyl-V™; Pfizer, Brandon, MB, Canada; 5 mg/kg body mass) was administered subcutaneously 30 min before surgical procedures. Both surgery and sham-operated animals were anesthetized using isoflurane (4%) and received local anesthesia applied over an incision site (2–5 mg/kg) using a 50/50 lidocaine (Xylocaine^®^; AstraZeneca, London, United Kingdom)/bupivacaine (Marcaine E 0.5% with Epinephrine 1:200,000 Vial; Pfizer, Brandon, MB, Canada) solution. Subsequently, a posterior midline incision through the skin and subcutaneous tissue was made parallel to L2–L6 spinous processes of the vertebrae and a left-side lumbar muscle (multifidus muscle) was resected to expose the lumbar facet capsule enveloping the lumbar segments L4–L6. In the surgery group, the left L4–L6 facet joints were compressed using Friedman micro rongeurs, whereas in the sham-operated group, the left L4–L6 facet joints were surgically exposed but not compressed. All muscles were sutured (braided 4-0 coated vicryl; Ethicon US; Cincinnati, OH, USA) and the skin closed using stainless-steel staples. After regaining consciousness, rats were returned to their cages and maintained in the same conditions as before surgery.

### Ultrasound scanning and image analysis

**Figure 1.**
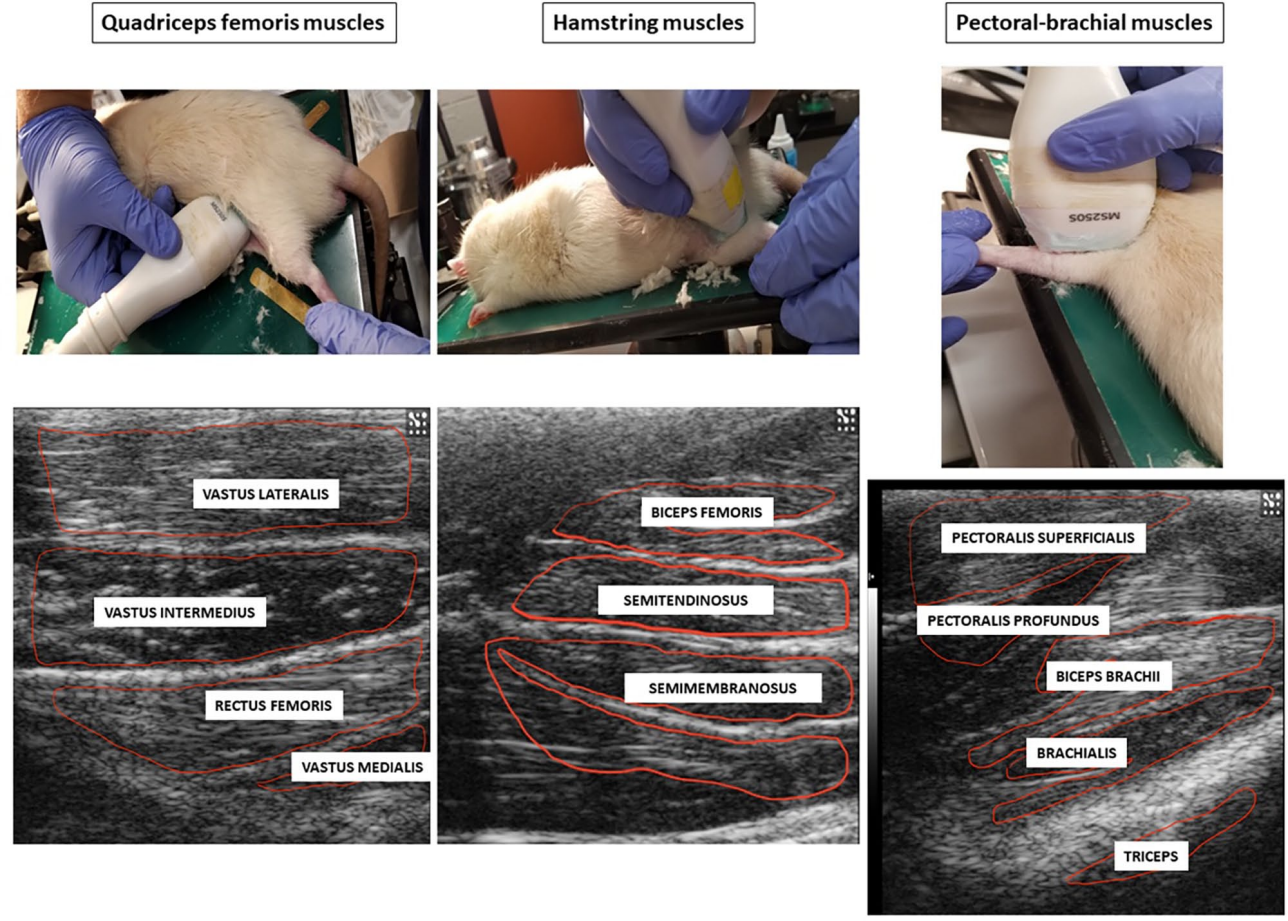
Photographs illustrating the scanning technique of pectoral-brachial, quadriceps femoris and hamstring muscles of rats, and corresponding ultrasonograms with delineated individual muscles used for echotextural image analyses. (A color version of this figure is available in the online journal.)

Ultrasound images were taken using the Vevo 2100 Visuals Sonic Ultrasound Diagnostic Medical Imaging System (FUJIFILM VisualSonics, Inc.; Toronto, ON, Canada). The anesthetized rats (4% isoflurane) were placed in the ventral position and examined with the variable frequency (13–24 MHz) MS250 linear-array probe at the left front and hindlimb regions (Figure 1). The equipment settings for frequency (24 MHz), overall and near/far gain, contrast, and focal points were kept constant throughout the entire study. All rats were subjected to ultrasonographic imaging of the quadriceps muscles (rectus femoris, vastus lateralis, vastus medialis, and vastus intermedius), hamstring muscles (semitendinosus, semimembranosus, and biceps femoris), and pectoral-forelimb muscles (pectoralis superficialis, pectoralis profundus, biceps brachii, brachialis, and triceps) prior to surgery (day 0) and on days 7, 14, and 21 following the procedures. We chose to include the pectoral-forelimb muscles as a neurosegmental control muscle in our study, as these muscles would not be amenable to neurogenic inflammatory mechanisms post-surgery, owing to the absence of a neurosegmental link to the primary pathology in the spine (L4–L6).

All scanning sessions were originally recorded as *.avi digital images. To analyze the muscle echotexture, selected still images of the muscles were converted to grayscale (with a bit depth of 8 ranges from 0 (absolute black) to 255 (absolute white)) using ImageProPlus^®^7.0 analytical software (Media Cybernetics Inc., Rockville, MD, USA). For each muscle analyzed in this study, a polygonal tool was used to delineate an area of interest containing the largest cross-sectional area of the muscle while excluding reflection artifacts, apparent blood vessels, epimyesium, bones, and tendons (Figure 1). Next, the first-order echotextural variables, namely, mean pixel intensity (numerical pixel values) and heterogeneity (mean standard deviation) of numerical pixel values, were recorded for each muscle at each time point.

### Pain behavior assessments

To confirm the occurrence of pathophysiological and sensory alterations associated with neurogenic inflammation,^21^ the mechanical and thermal sensitivity tests were performed on day 21 post-surgery. Thermal sensitivity of rats was assessed using a hot plate apparatus (Model 58725, Stoelting Co., Wood Dale, IL, USA). Animals were placed on a plate (22 × 22 cm) heated up to 50 °C (±2 C), and a withdrawal latency (time in seconds) was recorded. The withdrawal latency was defined as the time taken by each animal to exhibit first heat-avoidance responses such as jumping or licking their hindpaws, regardless of the paw side. If no response was observed during the 30 s of testing, the animal was removed from the plate and a 30-s latency was regarded as the withdrawal latency.^22^ Mechanical threshold was determined with an electronic von Frey (EVF) apparatus (IITC Life Science Inc., #2390 series, Woodland Hills, CA, USA). A non-noxious stimulus was applied by the probe onto the left hind paw of each rat and a positive response was regarded as a reflex withdrawal of the paw; five consecutive readings were obtained.^23^ The system scores and displays test readings in grams, based upon the amount of pressure applied.

### Western blots of inflammatory regulators

All animals were euthanized with carbon dioxide on day 21 of the experiment. Western blot analysis of inflammatory regulators was performed on muscle homogenates using methods described previously by Musumeci et al.^24^ Muscle samples (rectus femoris and biceps brachii, each 20–30 mg) were frozen and homogenized with cell lysis buffer (NP40 CLB-FNN0021; Thermo Scientific Fisher, Canada) containing protease inhibitor and serine protease inhibitor phenylmethylsulphonyl fluoride (PMSF). A copper-based assay using bicinchoninic acid (BCA) protein detection kit (Pierce BCA Protein Assay; Thermo Scientific, Canada) was used to determine protein content in the homogenized samples.^25^ Equal amounts of muscle sample proteins (15 μg) were separated by sodium dodecyl sulfate-polyacrylamide gel (15%) electrophoresis and transferred for 1 h at 0.2 A onto a polyvinylidene difluoride membrane. Membranes were blocked in 5% non-fat skim milk tris-buffered saline (TBS-T) containing 0.1% Tween-20 for 1 h at room temperature followed by an overnight incubation at 4°C with primary antibodies diluted in 5% bovine serum albumin. The primary antibodies used were as follows: anti-substance P (SP) antibody at 1:500 (orb215527), anti-calcitonin-gene-related peptide (CGRP) 1:250 (orb182870), and anti-PAR2 antibody at 1:500 (orb385619); Biorbyt Ltd., San Francisco, CA, USA; anti-proline-directed kinases (ERK1/2) antibody at 1:1000 (#9102); and anti-Ca²^+^/calmodulin-dependent protein kinase II (CaMKII) antibody at 1:1000 (#4436); Cell Signaling Technology, Denver, MA, USA. After three washes in TBS-T, the membranes were incubated at room temperature for 1 h with the secondary antibody (anti-rabbit – 1:2000, A0545; Sigma-Aldrich, Oakville, ON, Canada). Protein bands were detected using enhanced chemiluminescence (PerkinElmer; Waltham, MA, USA) and relative protein content was quantified by densitometry using a chemiluminescence detection system (Alpha Innotech Fluorchem HD2, Fisher Scientific, Hampton, NH, USA); all results were normalized against the loading control (alpha-tubulin protein, ab7291; Abcam, Cambridge, MA, USA).

### Statistical analyses

All data were analyzed using SigmaPlot^®^ statistical software (Systat Software Inc., San Jose, CA, USA). Two-way repeated-measures analysis of variance (RM-ANOVA) using independent variables of intervention (surgery vs sham-operated) and Time was performed to assess for differences in each of the dependent echotextural variables (mean pixel intensity and heterogeneity) for specific muscles within each muscle group (quadriceps, hamstring, and pectoral-brachial muscles). Two-way ANOVA using the independent variables of Muscle and Time was performed to test for differences in the dependent inflammatory mediator variable was used to compare relative concentrations of inflammatory mediators measured in rectus femoris and biceps brachii muscles of all rats. One-way ANOVA using Intervention (surgery vs sham-operated) as the independent variable was performed to test for differences in the quantitative behavioral responses (hot plate and von Frey tests) between the two groups of rats. Post hoc individual comparisons were done using the Tukey test (post-ANOVA) to determine the significant statistical differences between individual mean values (95% confidence interval). Differences between the two groups of animals for all single time-point observations were analyzed by Student *t*-test; whenever the data failed the normality (Shapiro–Wilk) and/or equal variance test, they were analyzed by the Mann–Whitney Rank Sum test. Correlations among echotextural, biochemical and behavioral variables were examined by a simple linear regression. All results are presented as mean ± standard error of the mean (SEM). Significance was set at an alpha of 0.05.

## Results

### Changes in first-order echotextural variables within each muscle group

#### Quadriceps femoris muscles

**Figure 2.**
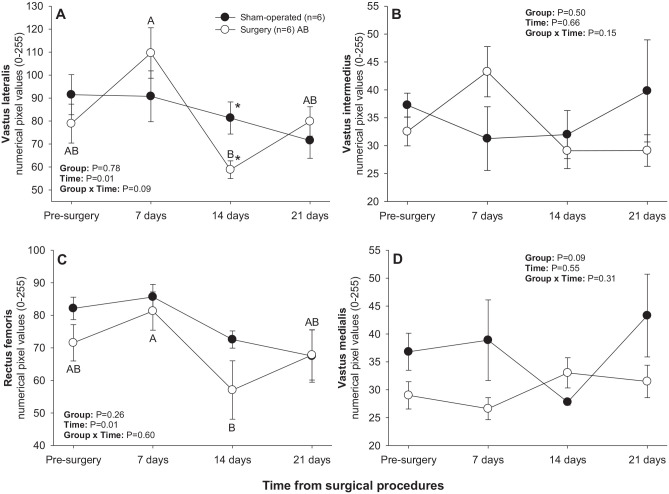
Mean (±SEM) numerical pixel values of quadriceps femoris muscles in sham-operated and experimental surgery (facet injury) rats examined ultrasonographically just prior to as well as 7, 14, and 21 days after surgical procedures. Within each group of animals, mean values denoted by different letters vary significantly (*P* < 0.05) and asterisks indicate significant differences between the two groups.

There was a significant main effect of Time for numerical pixel values of rectus femoris and vastus lateralis muscles (Figure 2). For both muscles (Figure 2(a) and (c)), there was a significant decline in mean pixel intensity between days 7 and 14 in the surgery group. In addition, mean pixel intensity values for vastus lateralis were greater (*P* < 0.05) in the sham-operated compared with surgery group on day 14 post-treatment (Figure 2(a)).

**Figure 3.**
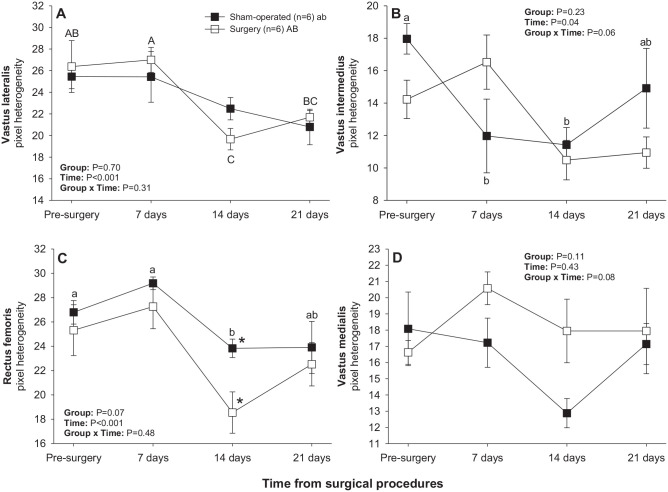
Mean (±SEM) values for pixel heterogeneity of quadriceps femoris muscles in sham-operated and experimental surgery (facet injury) rats examined ultrasonographically just prior to as well as 7, 14, and 21 days after surgical procedures. Within each group of animals, mean values denoted by different letters vary significantly (*P* < 0.05) and asterisks indicate significant differences between the two groups.

There was a significant main effect of Time for mean pixel heterogeneity values recorded in vastus lateralis, vastus intermedius, and rectus femoris muscles (Figure 3). A significant decline in pixel heterogeneity of vastus lateralis muscle from days 7–14 was observed in the surgery group (Figure 3(a)), whereas pixel heterogeneity of vastus intermedius and rectus femoris muscles declined from days 0–7 and from days 7–14, respectively, in the sham-operated group (Figure 3(a) and (c)). Sham-operated rats exceeded their surgery counterparts in pixel heterogeneity values for rectus femoris on day 14 after surgical procedures (Figure 3(c)).

#### Hamstring muscles

**Figure 4.**
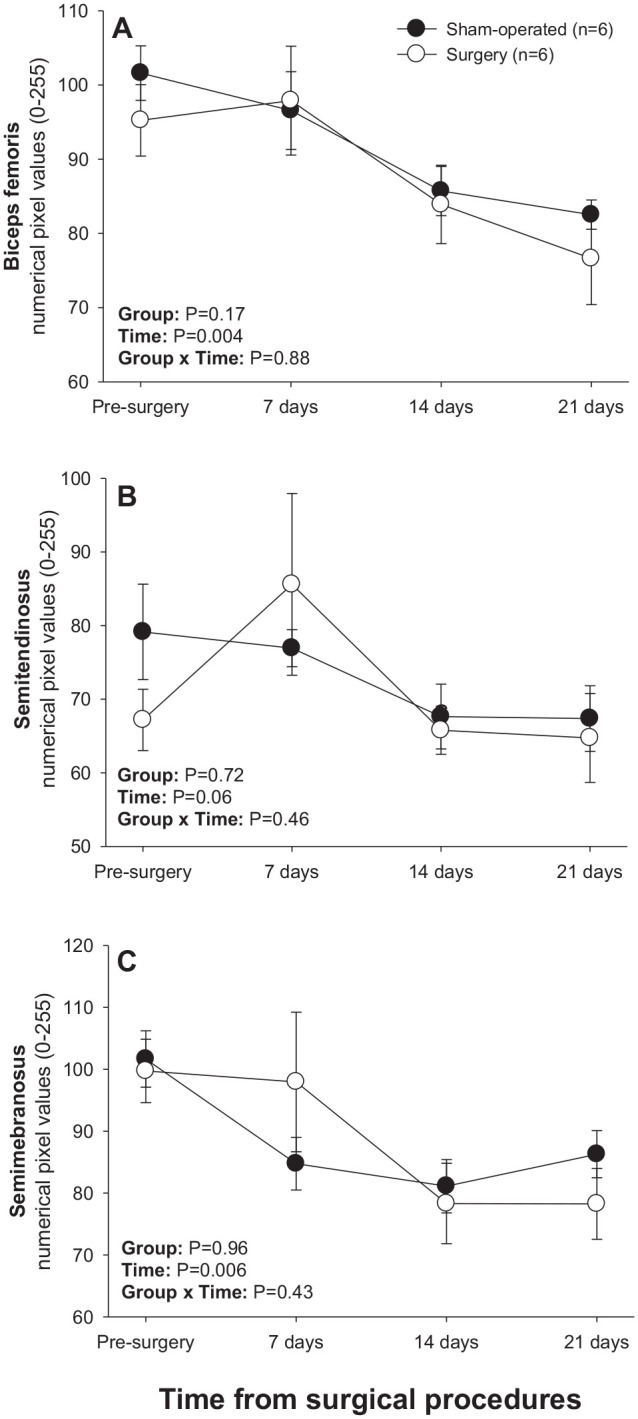
Mean (±SEM) numerical pixel values of hamstring muscles in sham-operated and experimental surgery (facet injury) rats examined ultrasonographically just prior to as well as 7, 14, and 21 days after surgical procedures.

**Figure 5.**
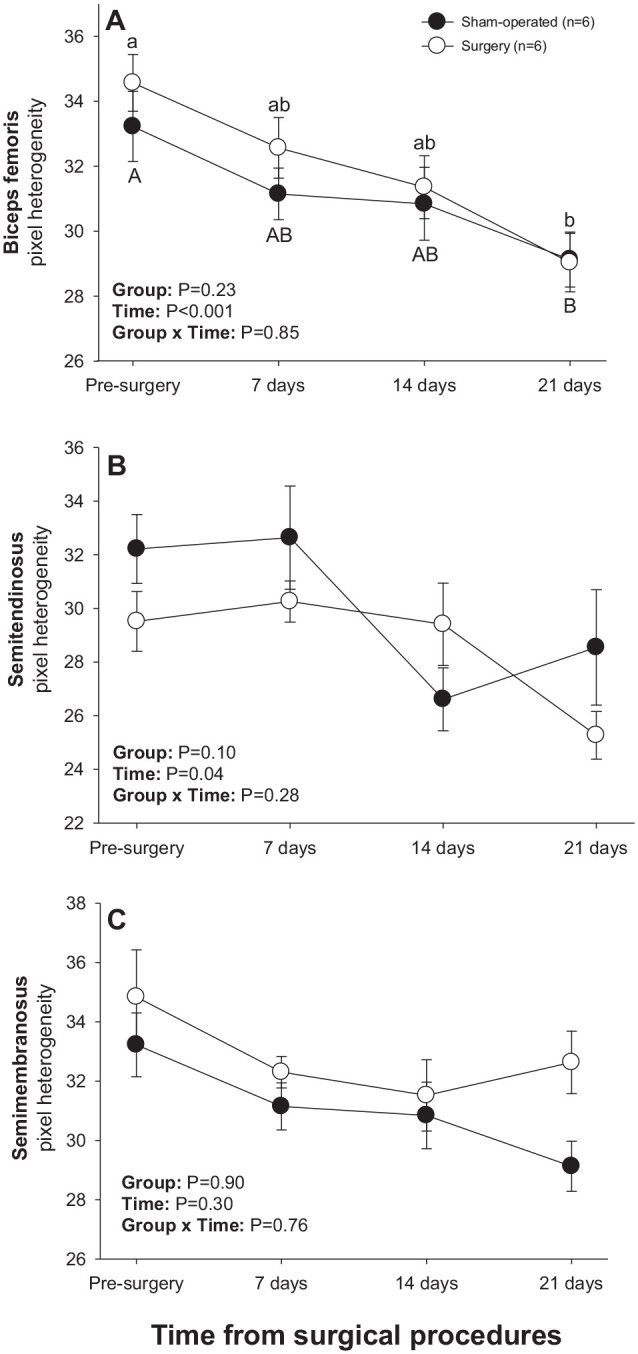
Mean (±SEM) values for pixel heterogeneity of hamstring muscles in sham-operated and experimental surgery (facet injury) rats examined ultrasonographically just prior to as well as 7, 14 and 21 days after surgical procedures. Within each group of animals, mean values denoted by different letters vary significantly (*P* < 0.05).

There was a significant main effect of Time for numerical pixel values in biceps femoris and semimembranosus muscles, but post-ANOVA comparisons did not reveal significant differences in mean values over time or between two subsets of rats studied (Figure 4). A significant main effect of Time was noted for pixel heterogeneity values of biceps femoris (Figure 5(a)); mean pixel heterogeneity values decreased (*P* < 0.05) from just before surgery to day 21 post-treatment in both groups of animals.

#### Pectoral-brachial muscles

**Figure 6.**
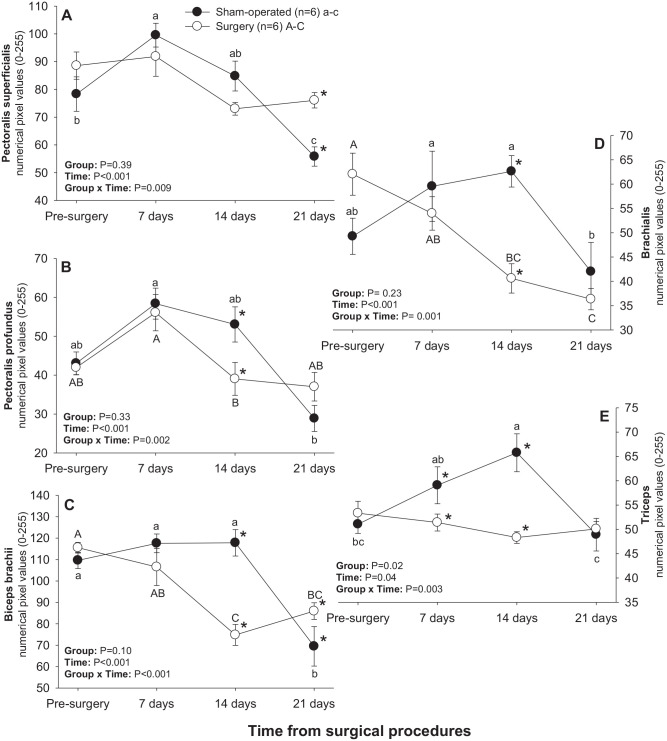
Mean (±SEM) numerical pixel values of pectoral-brachial muscles in sham-operated and experimental surgery (facet injury) rats examined ultrasonographically just prior to as well as 7, 14, and 21 days after surgical procedures. Within each group of animals, mean values denoted by different letters vary significantly (*P* < 0.05) and asterisks indicate significant differences between the two groups.

Significant main effects of Time and Time × Group interactions were observed for numerical pixel values of all brachial muscles scanned (Figure 6). A significant main effect of Group was recorded for numerical pixel values of triceps muscle. In sham-operated rats, mean numerical pixel values declined (*P* < 0.05) in all brachial muscles from days 14–21 post-treatment except for pectoralis profundus (Figure 6(b)), for which a decline in numerical pixel values during that interval was only numerical. In addition, numerical pixel values of pectoralis superficialis and triceps muscles rose (*P* < 0.05) 7 and 14 days after experimental procedures in the sham-operated group, respectively (Figure 6(a) and (e)). In the surgery group, numerical pixel values of pectoralis profundus and biceps brachii declined (*P* < 0.05) from days 7–14, and of brachialis muscle from days 0–21 post-surgery (Figure 6(b), (c), and (d)). For all muscles in this group except for pectoralis superficialis, mean numerical pixel values were greater in sham-operated compared with surgery rats on day 14, and numerical pixel values of the triceps muscle were also greater (*P* < 0.05) in sham-operated rats on day 7 following experimental procedures (Figure 6(e)). Surgery rats exceeded (*P* < 0.05) their sham-operated counterparts in mean numerical pixel values of pectoralis superficialis (Figure 6(a)) and biceps brachii (Figure 6(c)) on day 21.

**Figure 7.**
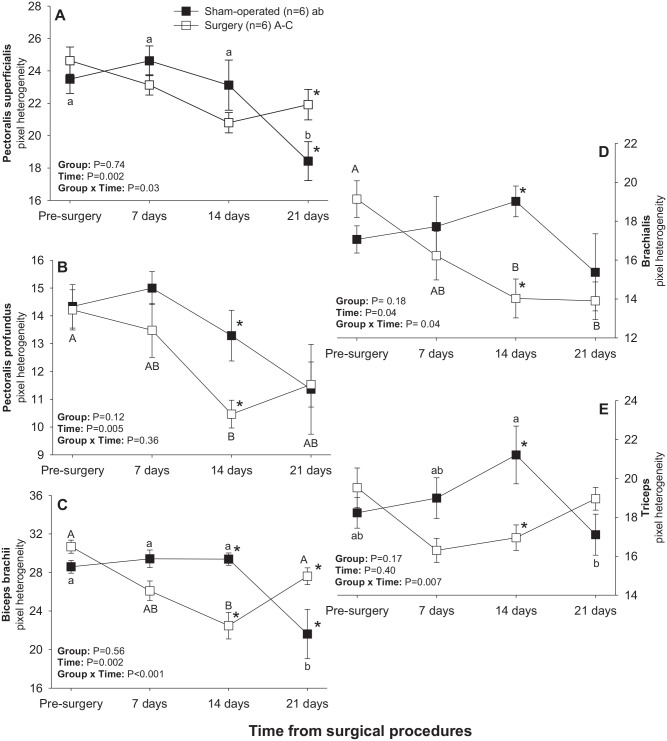
Mean (±SEM) values for pixel heterogeneity of pectoral-brachial muscles of sham-operated and experimental (facet injury) rats examined ultrasonographically just prior to as well as 7, 14, and 21 days after surgical procedures. Within each group of animals, mean values denoted by different letters vary significantly (*P* < 0.05).

There was a significant main effect of Time for mean pixel heterogeneity values in all brachial muscles except for triceps and a significant Group × Time interaction for all pectoral-brachial muscles excepting pectoralis profundus (Figure 7). Mean pixel heterogeneity values for pectoralis superficialis, biceps brachii and triceps muscles all declined (*P* < 0.05) between days 14 and 21 in sham-operated rats (Figure 7(a), (c), and (e)), whereas pixel heterogeneity of pectoralis profundus, biceps brachii and brachialis muscles decreased (*P* < 0.05) from days 0–14 in the surgery group (Figure 7(b), (c), and (d)). Apart from the pectoralis superficialis, all other muscles exhibited higher (*P* < 0.05) pixel heterogeneity values in sham-operated than in surgery rats 14 days after treatment. Mean pixel heterogeneity of pectoralis superficialis and biceps brachii was greater (*P* < 0.05) in surgery compared with sham-operated animals on day 21 (Figure 7(a) and (c)).

### Behavioral pain assessments

Induction of central sensitization was validated using thermal and mechanical sensitivity testing at day 21 post-intervention. Surgery animals demonstrated enhanced pain sensitivity to both tests. There was a 2.5-fold increase in thermal threshold of surgery compared with sham-operated rats on day 21 after surgical procedures (Table 1). The animals exposed to lumbar facet injury also showed increased (*P* < 0.05) tactile sensitivity (mechanical threshold) at the ipsilateral hindpaw compared with sham-operated rats 3 weeks post-surgery (Table 1).

**Table 1.** Comparisons of the thermal (hot plate latency) and ipsilateral (left hind paw) mechanical threshold values (electronic von Frey test) between sham-operated and experimental surgery (lumbar L4–L6 facet injury) groups of rats determined 21 days after surgical procedures.

Behavioral testing	Sham-operated (*n* = 6)	Surgery (*n* = 6)
Hot plate latency (s)*	8.4 ± 1.2^a^	21.4 ± 3.3^b^
Electronic von Frey (g)	20.0 ± 3.2^a^	53.9 ± 3.5^b^

* Mann–Whitney Rank Sum test.

ab *p* < 0.05 (within rows).

### Inflammatory mediators

There were no significant differences (*P* > 0.05) in the relative protein concentration of inflammatory regulators in non-neurosegmentally linked biceps brachii between sham-operated and surgery rats (Table 2). However, Western blot analyses revealed an occurrence of a 2.4-, 2.7-, and 1.9-fold rise in CaMKII, ERK1/2, and substance P concentrations, respectively, in neurosegmentally linked rectus femoris of surgery rats compared with sham-operated animals. No effect of facet injury on the relative expression of CGRP or PAR2 in the rectus femoris was noted in the rats of the present study (*P* > 0.05; Table 2). In surgery rats, muscle homogenate contents of all inflammatory regulators except for CaMKII were greater (*P* < 0.05) in rectus femoris than biceps brachii, whereas in sham-operated rats such a difference was only seen for ERK1/2, PAR2, and substance P (Table 2).

**Table 2.** Western blotting results of the relative expression of inflammatory regulators determined in left biceps brachii and rectus femoris homogenates collected post-mortem 21 days after experimental procedures in sham-operated and experimental surgery (lumbar L4–L6 facet injury) rats.

Inflammatory regulators	Sham-operated (*n* = 6)		Surgery (*n* = 6)	
	Biceps brachii	Rectus femoris	Biceps brachii	Rectus femoris
CaMKII	2.7 ± 0.4	1.6 ± 0.1^a^	3.4 ± 0.7	3.9 ± 0.4^b^
ERK1/2	0.60 ± 0.16^A^	1.8 ± 0.2^a B^	0.93 ± 0.29^A^	4.8 ± 0.5^b B^
CGRP	0.51 ± 0.07	1.1 ± 0.08	0.54 ± 0.02^A^	1.5 ± 0.5^B^
PAR2	0.43 ± 0.07^A^	0.76 ± 0.06^B^	0.45 ± 0.08^A^	0.97 ± 0.15^B^
Substance P	0.56 ± 0.03^A^	1.6 ± 0.2^a B^	0.63 ± 0.09^A^	3.0 ± 0.3^b B^

CaMKII: Ca²^+^/calmodulin-dependent protein kinase II; ERK1/2: extracellular signal-regulated protein kinase; CGRP: calcitonin-gene-related peptide; PAR2: protease-activated receptor 2.

Within rows, values denoted by different letter superscripts vary significantly (*P* < 0.05): ^ab^between groups and ^AB^between biceps brachii and rectus femoris within each group of animals.

### Correlational analyses

Numerical pixel values and pixel heterogeneity of biceps brachii were moderately and positively correlated with CGRP expression (*r* = 0.65 and *r* = 0.66, respectively; both *P* = 0.02). No other correlations among echotextural variables and the levels of inflammatory regulators were recorded for biceps brachii and no correlations for rectus femoris muscle of the present experimental rats. There were no quantitative correlations between first-order echotextural variables of either muscle and numerical scores obtained with the hot plate latency or von Frey testing. The expression of CaMKII and ERK1/2 in rectus femoris was directly related to numerical results of the hot plate latency tests (*r* = 0.67, *P* = 0.02 and *r* = 0.62, *P* = 0.03, respectively). The expression of CaMKII, ERK1/2, and substance P in rectus femoris was positively correlated with the results of electrical von Frey tests (*r* = 0.79, *P* = 0.002; *r* = 0.81, *P* = 0.001; and *r* = 0.66, *P* = 0.02). No significant correlations with pain sensitivity assessments were recorded for the levels of inflammatory mediators measured in the biceps brachii of surgery and sham-operated rats.

## Discussion

The overall objective of this study was to investigate quantitative changes in first-order echotextural variables (mean pixel intensity and heterogeneity) of skeletal muscles after experimental induction of spine osteoarthritis at the L4–L6 spinal levels. To our knowledge, no one has previously explored these changes in skeletal muscles using computer-aided ultrasound image analysis following experimentally induced spine osteoarthritis. Our findings support our primary hypothesis that, after the facet injury, mean numerical pixel values and pixel heterogeneity in the neurosegmentally linked quadriceps muscle (L4–L6) will show significant fluctuations over time. The ultrasound image analysis and statistical comparisons in the rats of the present study revealed that there was a decline in pixel intensity of the quadriceps femoris muscles with the highest numerical pixel values (i.e. rectus femoris and vastus lateralis) in the surgery group. In contrast, the mean pixel intensity of vastus medialis and vastus intermedius did not vary significantly over time.

The vastus lateralis is the strongest of the quadriceps muscles; it is estimated to contribute approximately 40% of the overall strength of the group.^26^ Moreover, it has been shown that after vastus lateralis paralysis caused by cutting the branch of the quadriceps nerve, the rectus femoris muscle mass increases significantly but the masses of vastus medialis and vastus intermedius remain unchanged.^27^ It is, therefore, feasible that there exists histomorphological and histophysiological similarity between rectus femoris and vastus lateralis. In fact, a detailed analysis of skeletal muscle microstructure in rats revealed that rectus femoris and vastus lateralis contained a greater proportion IIB (fast-twitch) and IID/X (heavy-chain myosin containing) muscle fibers but fewer type I (slow-twitch) and IIA fibers compared with vastus intermedius.^28^ The vastus intermedius also exhibited significantly higher citrate synthase activity (CSA) compared with other quadriceps femoris muscles.^28^ “The different types of mammalian muscle fibers are not only unique in their myosin heavy-chain composition but also have relatively distinct physiological and biochemical properties.”^28^ The latter could explain, at least to some extent, the differences in echotextural characteristics among individual muscles as well as varying echotextural changes inflicted by facet injury in surgery rats.

Muscles with abundant motor units innervating multiple sarcomeres may experience stronger chronic pain and neuroinflammatory impairments to their structure and function compared with their less innervated counterparts.^29^ The degree of innervation could potentially contribute to the degree of echotextural changes in skeletal muscles of the rats in this study, both prior to and after facet injury. With regard to radicular innervation of hindlimb muscles of the rat, ventral spinal roots L2–L5 supply knee extensors (e.g. quadriceps femoris), whereas L3–L5 are for knee flexors (e.g. biceps femoris and semitendinosus). Dorsal roots (afferent nerves) generally match the organization and distribution of the efferent nerves.^29^ However, when evaluated electrophysiologically, the radicular supply of some hindlimb muscles in the rat was found to be more extensive than that estimated anatomically.^29^ More studies are needed to corroborate a potential influence of muscle innervation on the alterations in their morphology and echointensity during the central neurogenic inflammation.

We have also observed significant changes in echotextural variables of quadriceps femoris muscles in the sham-operated group and in some pectoral-brachial region muscles of both sham-operated and surgery animals. Klahsen *et al.*^30^ have recently observed that facet degeneration and neuromyopathic changes can also occur, to a previously unrecognized extent, in sham-operated rats; that would explain the occurrence of echotextural changes in some quadriceps femoris muscles of sham-operated animals.^30^ Considering the echotextural changes in the non-neurosegmentally linked brachial myotome, we speculate that these alterations can be attributed to shifting the center of gravity of operated animals. It has been shown that the neonatal spinal transected rats develop autonomous weight-supported stepping as adults; the hind limbs of these rats bore significantly less weight than intact rats’ hind limbs (37% body weight on hindlimbs, 63% on forelimbs)^31^. Webb and Muir^32^ studied the locomotor ability of rats with unilateral thoracic or cervical spinal cord injury. They discovered that the position of the body’s center of gravity changes depending on the location of the spinal cord injuries. Cervical spinal cord injury rats carried more weight on their hindlimbs than controls and their center of gravity was more caudally positioned, whereas thoracic spinal injury animals carried more weight on their forelimbs than controls and their center of gravity was more cranially located. They concluded that rats could establish a general compensatory response to unilateral central nervous system injuries, which may aid in the animals’ locomotor stability.^32^ As the forelimbs are overloaded, compensatory adjustments may alter the frequency of neuromuscular unit firing, biochemical composition, and/or muscle fiber type.^31^ That in turn may alter the ultrasonographic appearance of the muscle.

Our findings do not support the null hypothesis that significant correlations exist between first-order echotextural variables and neuropeptides (substance P, CGRP) or pro-inflammatory markers (PAR2, ERK1/2, CaMKII). Our experimental facet injury model successfully induced central sensitization and neurogenic inflammatory responses within neurosegmentally linked lower limb muscles. This is evidenced by the fact that we observed significantly increased thermal and mechanical pain sensitivity in the hindpaw of surgery animals when compared with sham-operated controls. This is consistent with dysesthesia associated with central sensitization. Similarly, robust neurogenic inflammatory responses were observed within the neurosegmentally linked rectus femoris muscle when compared with biceps brachii in animals exposed to experimentally induced facet injury, but not sham-operated animals. This, in turn, is evidenced by our data showing significantly increased levels of substance P, ERK1/2, and CaMKII in the neurosegmentally linked rectus femoris muscle of surgery animals when compared with sham-operated rats; no differences in these biomarkers were observed between surgery and sham-operated groups in the non-segmentally controlled biceps brachii.

Several sensory neuropeptides released from the peripheral afferent terminals in the skeletal muscle are involved in neurogenic inflammation.^4^ Substance P is a neurotransmitter that relays pain signals, causes vasodilation, and leads to mast cell degranulation.^33–36^ Extracellular signal-regulated kinases (ERK1/2) are a part of a signal transduction pathway that governs intracellular processes during muscle contraction, but also mediate inflammatory responses and control transcription factors engaged in muscle cell adaptation to stressful conditions.^37,38^ Ca^2+^ CaM-dependent protein kinases (CaMKs) generally perform a modulatory role in all functional aspects of skeletal muscle activity such as force production, metabolism and local microinflammation during muscle twitch or extensive physical exercise.^25,39,40^ Interestingly, the expression of these inflammatory mediators appears to be most closely associated with the behavioral alterations detected with pain threshold tests (hot plate latency and von Frey). Unfortunately, our present results also indicate that first-order echotextural characteristics of rectus femoris and biceps brachii muscles are poorly correlated with the amounts of inflammatory mediators measured in muscle parenchyma, which agrees with the conclusion of Fujikake *et al.*^17^ that physical changes during myositis (e.g. edema) are the main cause of changes in muscle echotexture. Based on earlier results using echointensity-banding analyses of ultrasonograms and our piloting studies of selective image segmentation, we speculate that a lack of correlations among pixel characteristics and the levels of inflammatory mediators may be due mainly to limitations of currently used whole-image echotextural analyses to detect such quantitative relationships.

Chronic muscle pain affects 11–24% of the world’s population, with the majority of individuals having musculoskeletal discomfort at some point in their lives.^41^ Those who suffer from chronic muscle pain typically report lower productivity and frequently have to shift occupations or cease working completely because of discomfort they endure.^42^ One of the primary stimuli of muscle pain is neurogenic inflammation resulting from lumbar facet joint osteoarthritis.^5^ Since the nerves that transmit information to and from the lower/hind limbs originate in the lower back and so any pressure on a spinal column nerve roots can cause back pain as well as trigger neurological inflammation in the neurosegmentally linked muscles.^5^ Treatment’s goal is to eliminate inflammation, restore muscle performance, reduce morbidity, and improve quality of life. Tailoring the treatment of facet pain to the intensity of neurogenic inflammation could aid in optimizing its efficiency. Moreover, early diagnosis and targeted therapy are critical because soft tissue injuries can become a source of chronic pain.^43^ A reliable and noninvasive approach for diagnosing and monitoring muscle inflammation is vital.

In conclusion, the findings of the present study suggest that ultrasound image analysis is a promising technique for monitoring skeletal muscles throughout the course of neurogenic myositis (neurosegmentally linked myotomes) and following habituation or central sensitization (non-neurosegmentally linked muscles) associated with facet injury/facet pain in the rat experimental model. The findings of this study will contribute novel insight into changes in ultrasonographic imaging reflecting morphologic changes in muscle associated with inflammatory muscle disease. These data could provide important direction to informing future diagnostic imaging techniques in the assessment of muscle inflammation. However, we failed to demonstrate the existence of quantitative relationships among first-order echotextural variables, pain behavioral testing, and inflammatory mediators in segmentally linked myotomes. Further research is required to identify additional causes of changes in skeletal muscle echotexture including the pectoral-brachial region muscles and sham-operated animals. Our future direction is to examine such causes and their quantitative correlations with echotextural tissue attributes.
